# Silymarin and Cancer: A Dual Strategy in Both in Chemoprevention and Chemosensitivity

**DOI:** 10.3390/molecules25092009

**Published:** 2020-04-25

**Authors:** Dominique Delmas, Jianbo Xiao, Anne Vejux, Virginie Aires

**Affiliations:** 1 Université de Bourgogne Franche-Comté, F-21000 Dijon, France; anne.vejux@u-bourgogne.fr (A.V.); virginie.aires02@u-bourgogne.fr (V.A.); 2 INSERM Research Center U1231—Cancer and Adaptive Immune Response Team, Dijon, Bioactive Molecules and Health research group, F-21000 Dijon, France; 3 Centre anticancéreux Georges François Leclerc Center, F-21000 Dijon, France; 4 International Research Center for Food Nutrition and Safety, Jiangsu University, Zhenjiang 212013, China; jianboxiao@yahoo.com; 5 Laboratoire Bio-PeroxIL“Biochemistry of the Peroxisome, Inflammation and Lipid Metabolism”—EA 7270, UFR Sciences Vie Terre Environnement (SVTE), 6 Bd Gabriel, F-21000 Dijon, France

**Keywords:** silymarin, silybin, chemopreventive, chemosensitizer, ABC transporter, cell cycle, metabolizing enzymes, intrinsic and extrinsic pathway

## Abstract

Silymarin extracted from milk thistle consisting of flavonolignan silybin has shown chemopreventive and chemosensitizing activity against various cancers. The present review summarizes the current knowledge on the potential targets of silymarin against various cancers. Silymarin may play on the system of xenobiotics, metabolizing enzymes (phase I and phase II) to protect normal cells against various toxic molecules or to protect against deleterious effects of chemotherapeutic agents on normal cells. Furthermore, silymarin and its main bioactive compounds inhibit organic anion transporters (OAT) and ATP-binding cassettes (ABC) transporters, thus contributing to counteracting potential chemoresistance. Silymarin and its derivatives play a double role, namely, limiting the progression of cancer cells through different phases of the cycle—thus forcing them to evolve towards a process of cell death—and accumulating cancer cells in a phase of the cell cycle—thus making it possible to target a greater number of tumor cells with a specific anticancer agent. Silymarin exerts a chemopreventive effect by inducing intrinsic and extrinsic pathways and reactivating cell death pathways by modulation of the ratio of proapoptotic/antiapoptotic proteins and synergizing with agonists of death domains receptors. In summary, we highlight how silymarin may act as a chemopreventive agent and a chemosensitizer through multiple pathways.

## 1. Introduction

The past decade has been marked by intense scientific interest—both from researchers and industry—in the use of compounds or micronutrients of natural origin and their potential effects on human health. These phytomolecules have cellular targets similar to those of the new drugs developed by pharmaceutical companies. Indeed, more than 1600 patents are currently reported relating to flavonoids and 3000 patents relating to polyphenols. Pleiotropic pharmaceutical activities are claimed in fields such as cancer, inflammation arthritis, cardiovascular diseases, auto-immune diseases, eye diseases and many other domains. Among micronutrients and plant-derived compounds, flavonolignans are a family of natural products present in plants, composed of a flavonoid moiety and a phenylpropanoid or lignan part that may contribute to new strategies to fight various modern pathologies, and thus participate in preventive strategies. In the mid-1970s, Michael Sporn was the first to define the term “chemoprevention” in a study reporting the preventive power of natural forms of vitamin A on epithelial carcinogenesis [1]. Today, chemoprevention refers to a strategy using natural or chemical substances to inhibit, reverse or delay the multistage process of carcinogenesis with relative nontoxicity to normal cells. Moreover, as very well explain Michael Sporn and Nanjoo Suh in their commentary in Nature Reviews Cancer in 2002: “Extension of the latency period of carcinogenesis—so that people can have a high quality of life before dying of another cause at an advanced age—is a highly desirable strategy for controlling cancer and extending lifespan, even if total cure of advanced malignancy cannot be achieved” [2]. In addition to this capacity to act as a chemopreventive agent, many molecules, in particular of natural origin, have the capacity to act as a chemosensitizer or a therapeutic adjuvant. Indeed, as very well define by Gupta and colleagues, chemosensitization is one strategy to overcome chemoresistance [3]. It based on the use of one drug to enhance the activity of another by modulating one or more mechanism of resistance. This role as a chemosensitizer is some time confusing since a chemosensitizer may be a compound without anticancer activity itself or may both exert a chemopreventive activity and a chemosensitizing effect. In fact, such compounds may be used to: (1) minimize the toxicity of conventional chemotherapy; (2) modulate the alteration of drug metabolism; (3) bypass intrinsic or acquired resistance mechanisms (reduction in the influx of anti-cancer agents and/or exacerbation of active drug efflux mechanisms), thus helping to increase the survival rate. One of the most promising sources of new anti-proliferative drugs is the plant kingdom, given that data from the Food and Drug Administration (FDA) showed that 40% of the approved molecules are natural compounds or inspired by them, of which 74% are used in anticancer therapy. Therefore, we and others have shown that the use of natural molecules such as polyphenols can modulate xenobiotic-metabolizing enzymes, as well as the pathways leading to the death of cancer cells, making it possible to exert a chemopreventive activity and to restore sensitization to anticancer agents as observed with an emblematic and very described polyphenol of grapevines, resveratrol [3,4,5]. In similar manner, other natural bioactive molecules may present a dual activity both of chemopreventive and chemosensitizing. Indeed, among the world of polyphenols, in particular flavonoid class, silymarin, the extract of milk thistle, *Silybum marianum* [L.] Gaertn. [Asteraceae], and its major active flavonolignan silybin (or silibinin), may constitute a candidate of choice to exert both a chemopreventive action against various cancer models and a chemosensitizing activity with many compounds to counteract chemoresistance.

**Figure 1:**
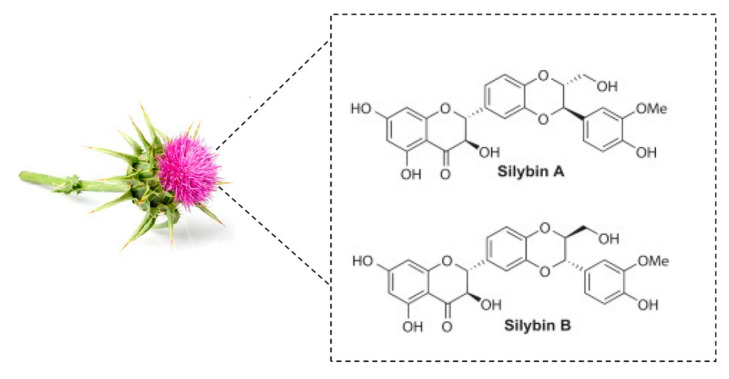
Major compounds of silymarin from Milk Thistle.

Silymarin has been used for more than 2000 years as a functional food ingredient for the treatment of a large number of liver disorders and silymarin is extracted from the seeds of milk thistle, *S. marianum* [L.] Gaertn. [Asteraceae]. Silymarin is a mixture of seven flavonolignans silybin A, silybin B, isosilybin A, isosilybin B, silychristin, isosilychristin, silydianin and one flavonoid, taxifolin, representing 65% to 80% of milk thistle extract and that can be determined by various HPLC separation techniques [6] (Figure 1). It is now used in Europe as complementary protection in patients receiving medication known to cause liver problems. The past five years have been marked by a revival of publications concerning silymarin, with more than 2670 citations in 2019 and a wide range of therapeutic properties have been proposed in the 1208 records for silymarin in the Web of Science including anti-oxidant, anti-inflammatory, anti-cancer and anti-viral activities, as well as its potential usefulness in the treatment of several liver disorders, such as chronic liver diseases, cirrhosis and hepatocellular carcinoma [7,8,9,10]. More specifically, silymarin and its derivatives may act on various targets involved in the development or the progression of cancer, this same targets may also be involved in its chemosensitizing properties [11,12,13,14].

The present review concentrate on the current knowledge on the potential targets of silymarin to highlight the different targets of silymarin that may be both in its preventive action but also sensitizing, with a parallel between the two mechanisms when it’s possible.

## 2. A Role for the Xenobiotics Metabolizing Enzymes (XME) Phase I and II in the Chemopreventive/Chemosensitivity Actions of Silymarin

### 2.1. Phase I Reactions

**Figure 2:**
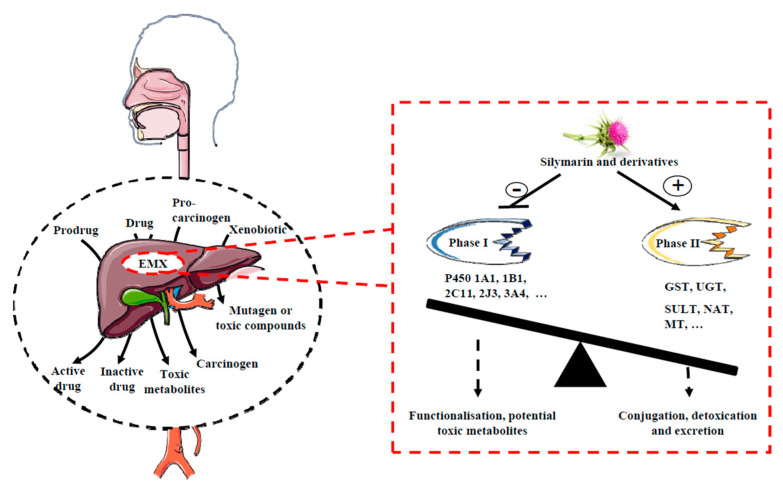
**Effect of silymarin on phase I and phase II enzymes.** Xenobiotics metabolizing enzymes (XME) biotransform various molecules such as prodrug, drug, procarcinogens, xenobiotics into active drug, inactive drug toxic metabolites, carcinogen and mutagen/toxic metabolites. Silymarin and derivatives may decrease the activity of phase I enzymes (i.e., P450) and activate phase II enzymes to increase the detoxication process. By these properties, silymarin and its derivatives may act as a chemopreventive or an adjuvant to decrease in normal cells toxicity-induced by chemotherapeutic agents.

The metabolism xenobiotics plays a considerable role for the transformations of xenobiotics in general whether it is the transformation of drug or prodrug into active drug, or toxic drug, nutrients or even of pro-carcinogens into carcinogenic proximal or into a final hydrosoluble metabolite. Biotransformations are catalyzed via specific cellular enzymes. At the subcellular level, these enzymes can be located in the endoplasmic reticulum, the mitochondria, the cytosol or the plasma membrane. A molecule can undergo several biotransformation reactions, some of which occur sequentially, and the metabolites can be very numerous. Functionalization reactions (called phase I) allow the creation of a functional group (e.g., hydroxyl) making the molecule sufficiently water-soluble to be eliminated (terminal metabolite) or capable of undergoing new chemical reactions (intermediate metabolite). More specifically, during phase I, organic xenobiotics can be transformed into a more hydrosoluble primary metabolite, usually by oxidation with mono-oxygenases. These enzymes are classified into two broad categories: those associated with cytochrome P450 and those associated with flavin adenine dinucleotide (FAD) or flavin adenine mononucleotide (FMN). More drugs and procarcinogens are able to induce cytochrome P450 enzymes (Figure 2).

### 2.2. Phase II Reactions

Conjugation reactions (called phase II) allow the combination of the drug or an intermediate metabolite with small endogenous polar molecules. The conjugate thus formed is water-soluble and eliminable.

Concerning phase II enzymes, they generally use the reactive group formed by phase I enzymes to form a water-soluble metabolite easily eliminated in biologic fluids. Conjugations realize the union of drugs or their metabolites with a conjugating agent originating from physiological metabolism. The product formed, called conjugate, is inactive and easily eliminated. The site of conjugation is essentially hepatic. There are six types, plus a few specific processes for certain substances. There are a wide variety of this type of enzymes, including glutathione-S-transferases (GST), UDP-glucuronosyltransferases (UGT), sulfotransferases (SULT), N-acetyltransferases (NAT) and methyltransferases (MT) (Figure 2).

### 2.3. Silymarin Exerts a Chemopreventive Action through an Inhibition of P450 Activity

Also thanks to its multiple properties, silymarin may play on the gene and protein expression of these enzymes by inhibiting functionalization enzymes (phase I), thus making it possible to protect against the generation of toxic compounds whether they originate from procarcinogens or from anticancer agents and may, on the contrary, activate phase II enzymes, thus allowing faster detoxification and thus eliminating potentially carcinogenic compounds or toxic compounds generated by chemotherapy (Figure 2, Table 1). Indeed, for example silymarin may exert a chemopreventive action against polycyclic aromatic hydrocarbon-induced carcinogenesis. The main P450 involves in the activation of polycyclic aromatic hydrocarbons into ultimate carcinogens is the P450 1A1 [15]. Silybin and dehydrosilybin inhibit basal and dioxin-inducible P450 1A1 catalytic activity in human keratinocytes (HaCaT) and human hepatoma cells (HepG2) where dehydrosilybin is a much stronger inhibitor than silybin (IC50 values were 22.9 ± 4.7 μmol/L and 0.43 ± 0.04 μmol/L, respectively) [16]. These results are in favor of a dehydrosilybin specificity towards 1A1 isoforms since other P450, (i.e., 2D6, 2E1 or 3A4) were inhibited only by much higher doses of dehydrosilybin [17]. When compared to other phenolic compounds such as protocatechuic acid, chlorogenic acid, tannic acid, it appears that protocatechuic acid, chlorogenic and silybin were more selective towards ethoxyresorufin O-dealkylase (EROD) (P450 1A1), methoxyresorufin O-dealkylase (MROD) (P450 1A2) and pentoxy-O-dealkylase (PROD) (P450 2B) in mouse liver microsomes from induced animals [18]. These activities reflect the activities for various isoforms of P450 (1A1, 1A2 and 2B, respectively), where silibinin inhibit in a non-competitive manner these three activities [18]. Moreover, silymarin protects Wistar rats against benzo(a)pyrene-induced damages by inhibiting P450 1A1 [19]. In other models slightly away from that cancer, these mechanisms are also found, where silymarin application abolished or suppressed the induction of P450 1A1 in liver, kidney and heart of the pyridine-treated Syrian hamsters [20]. Furthermore, others P450 play a role in the metabolism of many anticancer drugs, such as P540 3A4 for epipodophyllotoxins, ifosphamide, tamoxifen, taxol and vinca alkaloids and to produce various toxicities. For example, doxorubicin treatment induces various P450 such as CYP1A1, CYP1B1, CYP2C11, CYP2J3, CYP4A1, CYP4A3, CYP4F1, CYP4F4 and EPHX2 gene expression in the heart of DOX-treated rats. The consequences of these enzyme modulations is a modification of the level of their associated arachidonic acid metabolites in the heart of mal Sprague Dawley rats with a strong decrease in the cardioprotective 5,6-, 8,9-, 11,12- and 14,15-epoxyeicosatrienoic acids (EETs) explaining the progressive cardiotoxicity induced by doxorubicin [21]. Thus, by reducing the induction of these enzymes under the effect of certain anticancer agents, silybin and isosilybin may in fact reduce associated toxicities such as cardiotoxicity or nephrotoxicity. For example, silymarin was able to inhibit pregnane X receptor (PXR)-mediated CYP3A4 induction [22]. By computational molecular docking and by a LanthaScreen time-resolved fluorescence resonance energy transfer (TR-FRET) PXR assay, the authors have shown a strong interaction between both silybin and isosilybin and PXR suggesting that they may be suitable candidates to design potent PXR antagonists to prevent drug-drug interactions.

### 2.4. Silymarin Exerts a Chemopreventive/Chemosensitivity Action through an Activation of Phase II Enzymes

Conversely, silymarin may induce phase II enzymes to increase detoxification (Figure 2, Table 1). For example silymarin is able to reduce this induction and also to restore activity glutathione S-transferase (GST), glutathione reductase (GR) and glutathione peroxidase (GPO) [23] and by this way, silymarin prevents toxic effect of benzo(a)pyrene in Wistar rats by modulating GST, UGT, epoxide transferase or sulfotransferase [19]. The in vivo studies showed that silibinin at least partly counteracts the nephrotoxic side-effects of cisplatin [24,25]. It is an important point since cisplatin, which is a highly effective chemotherapeutic agent for a variety of cancers, including breast cancer, presents more side effects including genotoxicity, nephrotoxicity and acute myelotoxicity [26,27] and subsequently limits its use. In in vitro studies on human cancer cell lines, it may then be shown that the application of silibinin does not decrease the antitumor activity of either cisplatin or ifosfamide. Although some cell line-specific differences may exist, the available in vitro data do not indicate a significant interaction of clinically relevant levels of silibinin and the cytotoxic activity of these two major drugs used in testicular cancer [24,25].

### 2.5. Clinical Relevance of XME Modulation by Silymarin

Do these results obtained in vitro have real clinical relevance? There are currently very few reports studying the link between a silymarin-drug combination and the impact on XME activity. The study of van Erp et al., has shown on six cancer patients treated with irinotecan (dose, 125 mg/m^2^) given as a 90-min infusion once every week; four days before the second dose (patients received 200 mg milk thistle, thrice a day, for 14 consecutive days) that silybin concentrations after intake of milk thistle are too low to significantly affect the function of P450 3A4 and UGT1A1 in vivo, indicating that milk thistle is unlikely to alter the disposition of anticancer drugs metabolized by these enzymes [28]. In another context, silybin has shown no effect in healthy volunteers on the metabolism of indinavir, a protease inhibitor that is highly affected by induction or inhibition of P450 3A4 and that usually administrated to VIH patients [29,30].

Nevertheless, more studies should be done to determine if the silybin-drug interaction of these enzyme substrates is clinically relevant or not, and whether the very low doses of silymarin found physiologically can be at the origin of a modulation of EMX.

## 3. A Role for Phase III Transporters in the Chemopreventive/Chemosensitivity Actions of Silymarin

Generally, after functionalization or conjugation, phase III transporters are proteins that transport xenobiotics and their metabolites (active, inactive, toxic, carcinogen, etc.) through the membranes in order to eliminate them from the cell. Usually there are two main families of transporters: first, transporters of the solute carrier (SLC) family, most often expressed at the basolateral pole of the cell and which allow the entry of substances (influx transporters) into the cell. The most important in pharmacology are the “organic anion transporters” (OAT) and the “organic cation transporters” (OCT). Secondly, transporters of the ATP-binding cassettes (ABC) family, expressed either at the apical pole or at the basolateral pole of the cell and which function as efflux pumps, promoting the extrusion of drugs and toxins out of the cell (towards light or to the blood, depending on cell location). The most important in pharmacology are P-glycoprotein (P-gP) or multidrug resistance proteins 1 (MDR1), multidrug resistance-related protein (MRP) and breast cancer resistant protein (BCRP). These transporters use the energy of ATP hydrolysis for the unidirectional import or export of a considerable variety of substrates, from ions to macromolecules.

### 3.1. Silymarin and OATP

Concerning OATs, silibinin has been shown to inhibit OATP1B1, OATP1B3 and OATP2B1 in Chinese hamster ovary cells (Table 1) [31]. An inhibitory effect was also seen for MRP2. In contrast, the bile acid transporters Na^+^-taurocholate cotransporting polypeptide (NTCP) and bile-salt export pump (BSEP) were not affected by silibinin [31]. Similar results are obtained in human hepatocytes stably expressing OATP1B1, OATP1B3 and OATP2B1, where silymarin and silibinin significantly inhibited these OATPs [32]. Interestingly, authors indicate that calculation of the maximal unbound portal vein concentrations/IC_50_ values indicated a low risk for silymarin-drug interactions in hepatic uptake with a customary silymarin dose [32].

### 3.2. Silymarin and ABC Transporters

Concerning ABC transporters, various studies have shown the potential effect of this flavonolignan (Table 1). For example, silymarin at high dose (200 µM) was able to accumulate rhodamine 123 in Madin–Darby canine kidney II cells overexpressing the P-gP (MDCK-MDR1). Very interestingly in this study, silymarin promoted an increase on the intracellular of various chemical substrates of P-gP such as antiepileptic drugs [33,34]. Other transporters may be involved. Indeed, silibinin, in a dose-dependent manner with applying no cytotoxic effects, inhibited cell proliferation and reduced mRNA expression levels of some transporter genes, e.g., MDR1, MRP3, MRP2, MRP1, MRP5, MRP4, ABCG2, ABCB11, MRP6 and MRP7 in chronic myelogenous leukemia (CML) in vitro models, K562 and KCL22 cell lines [35]. These actions on various ABC transporters may thus modulate the efflux and bioavailability of various anticancer drugs.

Paclitaxel is one of the most important drugs used for the chemotherapeutic treatment of ovarian, mammary and non-small cell lung cancer [39]. Paclitaxel which is primarily metabolized in the liver by CYP3A4 and 2C8 and undergoes biliary excretion, present a low bioavailability after oral administration, which presents a major therapeutic problem. This explain by a poor solubility and first-pass metabolism which occurs into the liver and in the small intestine where paclitaxel is a substrate for P-gP in intestinal cells [40]. Several studies have highlighted the potential used of silymarin to modulate the bioavailability of paclitaxel. Indeed, a first study have shown in rats that after oral paclitaxel administration (40 mg/kg) in the presence of silibinin (0.5, 2.5 or 10 mg/kg), this flavonolignan significantly inhibited P-gP activity and compared to the control group, silibinin significantly increased the area under the plasma concentration-time curve (65.8–101.7% higher) of oral paclitaxel [36]. Silibinin also significantly increased (*p* < 0.05 by 2.5 mg/kg, 31.0% higher; *p* < 0.01 by 10 mg/kg, 52.9% higher) the peak plasma concentration of paclitaxel. Consequently, the absolute bioavailability of paclitaxel was increased by silibinin compared to that in the control group, and the relative bioavailability of oral paclitaxel was increased 1.15- to 2.02-fold [36]. These results are confirmed in a second study where oral bioavailability of paclitaxel in a Taxol^®^ formulation was enhanced in the combination with silymarin (10 and 20mg/kg) [41]. In similar manner as Sparreboom’s study, the mean maximum plasma concentration (C(max)) and the mean area under the plasma concentration-time curve (AUC(0-)(t)) of paclitaxel in the Taxol^®^ formulation were significantly increased 3-fold and 5-fold compared with control, respectively, following oral co-administration with 10mg/kg of silymarin [41].

Arsenic trioxide (As_2_O_3_) is highly efficient in treating acute promyelocytic leukemia [42], but it is limited in solid tumors as a single agent [43]. The silibinin action on arsenic sensitivity and accumulation may be associated with ABC transporter interaction. Indeed, silibinin increased the accumulation of arsenic more strongly and this accumulation may be attributed to a decreased rate of arsenic export [44].

Other ABC transporters and drugs can be involved such as MRP1 where silymarin increased accumulation of daunomycin and vinblastine in human pancreatic adenocarcinoma cell Panc-1 by inhibiting MRP1-mediated transport [38]. Silymarin accumulates also mitoxantrone in breast cancer resistance protein (BCRP/ABCG2)-overexpressing cell lines In two separate BCRP-overexpressing cell lines [45]. Similar drug such as doxorubicin was accumulated in multidrug resistant human breast cancer cell lines MCF-7 overexpressing P-gP. Consequently, silymarin potentiated doxorubicin cytotoxicity in these cells and inhibited P-gP ATPase activity [46].

In another context, but interesting, silibinin may inhibit the cytochrome P4503A4-mediated metabolism of loratadine in rats, resulting in reducing gastrointestinal and hepatic first-pass metabolism and the P-gP efflux pump in the small intestine, consequently, silibinin significantly enhanced the oral bioavailability of loratadine [37].

## 4. Cell Cycle and its Important Checkpoints for the Chemopreventive/Chemosensitivity Actions of Silymarin

**Figure 3:**
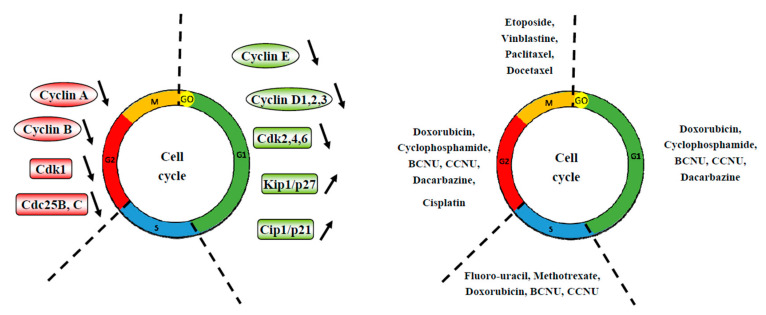
**Effect of silymarin on key regulators of cell cycle.** Cell cycle progression is controlled by key regulators such as cyclin and their kinase, cyclin-dependent kinase (Cdk). Silymarin and its derivative are able to block tumoral cells in the different phase of the cell cycle through a decrease of cyclins and associated Cdks and to increase the inhibitors proteins that negatively regulate the cell cycle. Furthermore, various antitumoral agents are able to target specific phase of the cell cycle. By this way, silymarin and its derivatives may sensitize cancer cells to these anticancer agents.

Thanks to its action at the level of the cell cycle, silymarin and its derivatives may also play a double role at the same time by (i) limiting the progression of cancer cells through the different phases of the cycle thus forcing them to evolve towards a process of cell death or by (ii) accumulating cancer cells in a phase of the cell cycle, thus making it possible to target a greater number of tumor cells with a specific anticancer agent (Figure 3, Table 2).

### 4.1. Silymarin and G0/G1 Arrest

Indeed, silibinin was able to block cell cycle arrest in G1 phase. This decreases involved key regulators of cell cycle such as cyclins D1, D3, E and their associated cyclin-dependent kinases, Cdk 2, 4, 6 in human of various cancer cells (i.e., prostate, hepatoma, colon, non-small cell lung cancer, epidermoid carcinoma, ovarian cancer, melanoma) [47,48,49,50,51,52,53,54,55,56,57,58,59,60,61,62]. Moreover, Cdk–cyclin complexes are negatively controlled by the Kip/Cip family of CDKIs, namely, Kip1/p27 and Cip1/p21, in addition to the INK family of CDK inhibitors (CDKIs) [63]. It appears that silibinin was also able to decrease the kinase activities of Cdk2 and 4 and at the inverse to increase Cdk inhibitors that control negatively Cdk-cyclin complexes such as Kip1/p27, Cip1/p21 and p18/INK4C in in vitro and in vivo in various cancers [47,48,49,50,51,52,54,55,56,57,64,65]. Subsequently, by acting on these key regulators, silibinin blocks cell cycle progression which is reinforced by a cytoplasmic sequestration of cyclin D1 and Cdk2 contributing to this G1 arrest and finally the cancer cell proliferation (Figure 3, Table 2). Similar results were obtained in some in vivo models, where silibinin decreased protein expression of cyclins A, B1 and E and their respective Cdks in a dose dependent-manner in transgenic adenocarcinoma of the mouse prostate where these cyclins are increased with progression in 20–30-week and 30–45-week positive controls [64]. Furthermore, silibinin causes hypophosphorylation of the retinoblastoma protein (pRb), a tumor suppressor by phosphorylation more particularly Rb-related proteins Rb/p107 and Rb2/p130 [52,59,65]. This silibinin action plays a main role in the disruption of cell cycle progression. Recently, a study has shown that silymarin-mediated cyclin D1 downregulation may result from proteosomal degradation through its threonine-286 phosphorylation via NF-κB activation in colon cancer cells [66].

### 4.2. Silymarin and G2/M Arrest

Another important point of control is the G2/M interface which is triggered by activation of Cdk1 linked to cyclin A then to cyclin B. The binding of Cdk1 to cyclin B allows passage from phase G2 to phase M. Cyclin B by binding to Cdk1 makes accessible a threonine at position 161 which is phosphorylated. Silymarin and silibinin were also able to block cell cycle in the G2/M phase where the key regulators of this phase, cyclins B1 and A where decrease as well as Cdk1 (so called Cdc2) and its phosphorylated forms (Tyr15) leading to an inhibition of its activity [47,58,60,62,67,68] (Figure 3, Table 2). This molecular mechanism also involves a decrease of Cdc25B and Cdc25C phosphatases with an increased phosphorylation of Cdc25C at Ser216 and its translocation from nucleus to the cytoplasm. This action leads to an increases binding with the protein 14–3-3 beta [47,49]. Subsequently, by their actions on this Cdc25 phosphatases, silymarin and silibinin prevent dephosphorylation and activation of Cdk2/cyclin A and Cdc2/cyclin B, and also, through Cdc25c action prevent dephosphorylation and activation of Cdc2/cyclin B mitotic kinase complex and thereby block cell entry into mitosis [47,48,49].

### 4.3. Silymarin and Chemosensitization through a G0/G1 or a G2/M Arrest

By this way, silymarin and its derivatives may sensitize tumor cells to anticancer agents targeting G0/G1 phase such as cyclophosphamide, arsenic, doxorubicin, BCNU, CCNU or dacarbazine (Figure 3, Table 2).

We have previously shown that silymarin may potentiate the action of arsenic trioxide through an inhibition of P-gP. But this drug may also present some toxicities. A combination of silibinin with 0.5 or 5 µM arsenic induced G1 or G2/M phase arrest, respectively, and decreased the protein levels of Cdk2, -4 and -6 and cyclin D1, D3 and E and increased CDK inhibitors p21 and p27 [69].

In another example, when baicalein was used in combination with silymarin on HepG2, an additive effect at 24 h and a synergistic effect at 48 h were observed to eradicated this hepatocarcinoma cells [70]. Combination of both drugs synergistically increased the percentages of cells in G0/G1 phase and decreased those in S-phase, which were associated with up-regulation of Rb, p53, Cip1/p21 and Kip1/p27 and down-regulation of cyclin D1, cyclin E, Cdk4 and phospho-Rb [70].

A combination of cisplatin or carboplatin with silibinin resulted in a stronger G2/M arrest, compared to these agents alone showing a moderate G2/M and G1 arrests in case of cisplatin and silibinin and a complete S phase arrest with carboplatin, respectively. A stronger G2-M arrest by these combinations was accompanied by a substantial decrease in the levels of cdc2, cyclin B1 and cdc25C [71]. Consequently, these combination induced a synergic effect to kill prostate cancer cell lines [71].

Silibinin also strongly synergized with doxorubicin to induces a strong G2/M arrest in cell cycle progression to induce cell death of prostate cancer (PCA) cells [72,73]. The underlying mechanism of G2/M arrest showed a strong inhibitory effect of combination on cdc25C, cdc2/p34 and cyclin B1 protein expression and cdc2/p34 kinase activity [73]. Interestingly, silibinin also synergized with doxorubicin, cisplatin or carboplatin in both estrogen-dependent and -independent human breast carcinoma, MCF-7 and MDA-MB468 cells [74]. The used of doxorubicin is limited by its inducing cardiotoxicity. Indeed, doxorubicin induces apoptosis and necrosis in healthy tissue causing toxicity in the brain, liver, kidney and heart [75]. Silibinin (60 mg/kg, orally) may show cardioprotective and hepatoprotective effects against doxorubicin-mediated toxicity (1.66 mg/kg, i.p.). Indeed, silymarin was able to prevent increase in AST and CK serum activity and myocardial excitability of rats caused by doxorubicin. It also significantly reduces doxorubicin-prooxidative activity and decreases histological changes in liver and heart tissue of animals treated with doxorubicin [76]. The protective effect of silymarin against doxorubicin seems also involved radical scavenging, cell membrane stabilization and iron chelation [77]. This hepatoprotective action is very interesting by in the same time when used in combination with doxorubicin, silibinin strongly synergized with it to induce G2/M arrest and growth inhibition and apoptosis of hepatocarcinoma cells [78]. More specifically, silibinin–doxorubicin combination inhibited cdc2/p34 kinase activity, moderately increased the expression of cdc25C-cyclin B1-cdc2/p34 associated upstream kinases (Chk1) [78].

In a study performed on human ovarian cancer lines, A2780s and PA-1 cells, it was found that silymarin effectively inhibited cell growth in a dose- and time-dependent manner and induced cell cycle arrest at G1/S phase both in A2780s and PA-1 cells [57]. Ovarian cancer SKOV-3 cells and paclitaxel-resistant ovarian cancer A2780 cells growth were considerably inhibited by paclitaxel and silibinin combination treatment by a G2/M arrest [79,80]. These mechanism involved a down-regulation of surviving and an induction of the two tumor suppressor genes up-regulation, p53 and p21 [79]. A similar G2/M arrest is observed in human gastric cancer cells [81] and in human prostate cancer cell lines [82] with this combination.

## 5. Extrinsic and Intrinsic Cell Death Pathways for the Chemopreventive/Chemosensitivity Actions of Silymarin

Induction of cell death pathways and more particularly of apoptosis also constitutes a pathway of choice in the context of chemoprevention to direct the excess of abnormal cells to death. The induction of apoptosis triggered by polyphenolic compounds has been observed in various cell types with different pathways [5]. Indeed, it has been demonstrated that polyphenols are able to activate cell death by the mitochondrial pathway or by the death receptor pathway. These molecules due to their capacity to induce the activation of the extrinsic and intrinsic death pathways can restore sensitivity to the agents targeting these pathways [83]. It is the case of silymarin and its derivatives.

### 5.1. Silymarin Modulates Mitochondrial and Death Receptors Pathways for its Chemopreventive Action

For example Bcl-2 which is a negative factors in term of patients survival, is decreased by silibinin in various cancer cells such as human breast cancer call (MCF-7, T47D) [84], glioma cells [85], ovarian cancer cells [57], in melanoma cells [58], in pharynx squamous cell carcinoma [86], in colon cancer cells [87] and in cervical cancer cells [88]. At the inverse the pro-apoptotic Bax protein is also upregulated and subsequently, capsase-3 activation [85]. These effects seem involved the PI3K pathway where silibinin inhibits PI3K reducing Forkhead box M1 (FoxM1) and subsequently, lead to activation of the mitochondrial apoptotic pathway [85].

Concerning extrinsic pathway, this way involves binding of a death ligand to a receptor of the tumor necrosis factor (TNF) superfamily [100] with its subsequent trimerization and recruitment of adaptor proteins (e.g., Fas-associated death domain (FADD), TNF receptor-associated death domain (TRADD)) to their cytosolic death domains. To date, the best characterized death receptors and their corresponding ligands are the FasL/FasR, TNFα/TNFR1 and tumor necrosis factor-related apoptosis-inducing ligand (TRAIL)/TRAILR1 or TRAILR2. Silibinin is also able to activate this apoptotic pathway by the up-regulation of TRAIL and TRAIL Death receptor 5 (DR5) transcripts and by the activation of caspase-3 and -8 in hepatocarcinoma cells [98], results that are confirmed in in vivo model [98]. In human colon cancer cell models, silibinin was also able to enhance the expression (protein and mRNA) of TRAIL death receptors (DR4/DR5) at the cell surface in colon cancer SW480 cells and to induce their expression in TRAIL-resistant SW620 cells normally not expressing DR4/DR5. Caspase-8 and -10 were activated and interestingly, the protein Bid was cleaved in SW480 cells indicating a cross-talk between extrinsic and intrinsic apoptotic pathway [99].

### 5.2. Silymarin Synergizes with Anticancer Drugs to Induce Apoptosis

By inducing these intrinsic and extrinsic pathways, silymarin may not only exert a chemopreventive action, but also may also reactivate these cell death pathways by modulating the ratio of proapoptotic/antiapoptotic proteins and by synergize with agonists of receptors for death domains.

Silymarin and paclitaxel may cooperate positively to induce a G2/M arrest [57,79,80,82]. It is not the only one action, indeed, silymarin/paclitaxel combination presents a synergistic effect to i) increase the Bax/Bcl-2 ratio by suppressing Bcl-2 gene expression into the prostate and gastric cancer cell lines [81,82]; ii) besides, they increased also the expression of TNFRSF10A, TNFRSF1A into the prostate cancer cells [82] and TNFR6 (Fas)/Fas ligand in gastric cancer cells [81] and thus inducing procaspase-8 and procaspase-3 activation and PARP cleaved. In similar manner, a silibinin/arsenic combination synergizes to induce apoptosis in human glioblastoma cell line [101]. The intrinsic apoptosis pathway may also be targeted by other combinations and mechanisms. Indeed, silibinin/platinum compound combinations were also effective in inducing apoptosis such as cisplatin, carboplatin and oxidovanadium complex when combined with silibinin enhanced apoptosis [71,102]. Apoptosis induction was further confirmed by PARP and caspases 3, 9 and 7 whose cleaved levels were also enhanced by combination treatment. In addition, there was a significant increase in cytochrome c release in the cytosol following treatment of DU145 cells with these combinations [71]. As previously, silibinin/doxorubicin combination that synergize to induce and arrest at G2/M phase of the cell cycle, was also able to synergize to enhance apoptosis in hepatoma cells [78,103], prostate cancer cells [73,104], breast cancer cells [74,105,106] and lung tumor cells [107]. Silibinin may also synergize with the ligands of death receptors such as TRAIL. Indeed, silibinin and TRAIL synergistically induced cell death in human colon adenocarcinoma cells through an up-regulation of death receptor 4 (DR4) and DR5 by silibinin [94]. The authors shown by using a human recombinant DR5/Fc chimera protein that has a dominant-negative effect by competing with the endogenous receptors abrogated cell death induced by silibinin and TRAIL, demonstrating the activation of the death receptor pathway. This synergistic action induces the cascade of death pathway through the activation of caspase-3, -8 and -9. Very interestingly, silibinin and TRAIL potentiated activation of the mitochondrial apoptotic pathway and down-regulated the anti-apoptotic proteins Mcl-1 and XIAP [94]. The molecular mechanism seems involved an increase of DR5 in a transcription factor CHOP-dependent manner and a down-regulation of the anti-apoptotic protein FLIP (L), FLIP(S) and surviving through proteasome-mediated degradation [108]. More recently, this properties has been used to sensitize the rhTRAIL-resistant triple-negative breast carcinoma cells to rhTRAIL-induced apoptosis through the up-regulation of death receptors 4 and 5 and the down-regulation of surviving transcriptionally [109]. Other death ligands are able to synergize with silibinin such as anti-Fas agonistic antibody CH11 that in combination with silymarin enhance cytotoxic effect in human malignant melanoma cells [110].

## 6. Clinical Studies of Silymarin and Derivatives in a Cancer Context

A certain number of clinical studies have been launched in phases 1, 2 and 3 in order to test the efficacy of silymarin and its various formulations in several types of cancer (Table 3); Silymarin seems safe in humans at therapeutic doses and is cell tolerated even at a high dose of 700 mg three times a day for 24 weeks [111]. In this way, two clinical trial have highlighted the protective effects of silymarin during chemotherapy. In a phase I clinical trial, it has been shown that an administration during 12 weeks of 800 mg/day of silymarin during the patient’s methotrexate and 6-mercaptopurine chemotherapy shown that patient had normal liver transaminase levels and there was no further interruption of therapy [112]. In a phase III clinical trial, silymarin was used in combination with soy, lycopene and antioxidants to delay prostate specific antigen progression after radiotherapy and prostatectomy in patients with prostate cancer [113]. Results of this study showed that the dietary supplement significantly improved the slope of 2log transformed PSA concentrations in comparison with placebo.

## 7. Conclusions

Silymarin, extracted from milk thistle seeds and its derivatives present a pleiotropic effect with multiple targets in the cancerous cells. This ability to modulate various proteins and genes makes silymarin a very good candidate for a chemopreventive action, thus enabling it to act on the key stages of carcinogenesis, namely initiation, promotion and tumor progression. These steps are controlled in particular by the balance of phase I and II metabolism enzyme, by the progression into the cell cycle and by the induction of programmed cell death, namely apoptosis. By these same mechanisms, silymarin can also act as a chemosensitizer by modulating the balance of phase I and II, by increased the action of anticancer drugs that target specifically cancer cells in a specific phase of the cell cycle and by restoring the induction of cell death pathways as well as intrinsic and extrinsic pathways. These different mechanisms and signaling pathways described in this review are not the only ones to be involved in the chemosensitization of anticancer agents by silymarin. Indeed, silymarin may cooperate positively with histone deacetylase (HDAC) inhibitors (i.e., trichostatin A), and with DNA methyltransferase inhibitor (i.e., 5′-Aza-deoxycytidine) in upregulating E-cadherin expression together in human non-small cell lung cancer cells to strongly decrease the invasion/migration of these cells [119].

Besides sensitization, silymarin may also play a very interesting role not by potentiating the action of anticancer agents but by reducing the toxic effects on vital organs or on healthy cells. For example, silymarin can activate or decrease various reactions such as spontaneous reduction of lipid peroxidation levels in the heart tissue where silymarin reduced creatine kinase isoenzyme MB (CK-MB), lactate dehydrogenase (LDH) and cardiac troponin I (cTnI) due to its antioxidant properties and consequently this flavonolignan was clearly efficient in protecting the cardiac myocytes from the toxic action of cisplatin [120]. In similar manner, silymarin significantly decreased adriamycin-induced acute cardiotoxicity with a reduction of myocardial MDA contents and also inhibited adriamycin-induced renal tubular damage in rats [121]

Due to these various properties and its potential benefits in chemoprevention and adjuvant in chemotherapy, much more studies are necessary to better understand the action mechanisms of silymarin and its derivatives. More particularly, it is important to better characterize the bioavailability and metabolites of silymarin or its derivatives in humans and to determine if these metabolites are themselves active on cancer cells either the metabolites produced can constitute a reserve of silymarin able to act on tumor cells. Other parameters must also be determined in the context of use as a therapeutic adjuvant, in particular determination of the administration sequence of silymarin or its derivatives. Indeed, preconditioning with silymarin may condition the organism to receive conventional anticancer agents and reduce the toxic effects on the renal and cardiac levels or may potentiate the effects of these drugs on tumor cells. Pretreatment with silymarin or its derivatives or co-treatment with therapeutic drugs is of great importance. These questions require numerous studies in order to be able to consider silymarin or one of its derivatives as a potential adjuvant therapeutic.

## Figures and Tables

**Table 1:** Targets in cellular and animal models of Milk Thistle’s Active Components.

	Compound	Target		Cellular or Animal Model	Reference
**Inhibition**	Silybin, dehydrosilybin	**Phase I enzymes**	Ethoxyresorufin O-dealkylase (EROD) (P450 1A1)	human keratinocytes (HaCaT), human hepatoma cells (HepG2)	[16]
	Silybin, silibinin		P450 1A1, methoxyresorufin O-dealkylase (MROD) (P450 1A2), pentoxy-O-dealkylase (PROD) (P450 2B)	mouse liver microsomes	[18]
	Silymarin		P450 1A1	Wistar rats, Syrian hamsters	[19,20]
	Silymarin		CYP3A4	LS180 colon adenocarcinoma cells	[22]
	Silibinin	**Phase III transporters**	Organic Anion Transporters (OAT)P1B1, OATP1B3, OATP2B1, MRP2	Chinese hamster ovary cells	[31]
	Silymarin and silibinin		OATP1B1, OATP1B3 and OATP2B1	human hepatocytes	[32]
	Silymarin		P-glycoprotein (P-gP)	Madin–Darby canine kidney II cells, MCF-7	[33,34]
	Silibinin		MDR1, MRP3, MRP2, MRP1, MRP5, MRP4, ABCG2, ABCB11, MRP6 and MRP7	K562 and KCL22 cell lines	[35]
	Silibinin		P-gP	Rats	[36,37]
	Silymarin		MRP1	human pancreatic adenocarcinoma cell Panc-1	[38]
	Silibinin	**Phase I enzymes**	cytochrome P4503A4	rats	[37]
**Activation**	Silymarin	**Phase II enzymes**	glutathione S-transferase (GST), glutathione reductase (GR), glutathione peroxidase (GPO)	mouse liver	[23]
	Silymarin		GST, UDP-glucuronosyltransferases (UGT), epoxide transferase, sulfotransferase	Wistar rats	[19]

**Table 2:** Targets in cellular and animal models of Milk Thistle’s Active Components used alone: cell cycle, extrinsic and intrinsic cell death pathways.

	Compound	Target		Cellular or Animal Model	Reference
**Inhibition**	Silibinin	**G1 phase**	cyclins D1, D3, E/cyclin-dependent kinases, Cdk 2, 4, 6	Human prostate, hepatoma, colon, non-small cell lung cancer, epidermoid carcinoma, ovarian cancer, melanoma cells	[47,48,49,51,52,57,58,59,60,62,89,90,91]
			cyclins A, B1 and E and their respective Cdks	Transgenic adenocarcinoma of the mouse prostate	[92]
			pRb	Human non-small cell lung cancer cell, apc (−/+) mice, human prostate carcinoma DU145 cells, human hepatoma HepG2 cells	[52,59,70,93]
	Silymarin and silibinin	**G2/M phase**	cyclins B1/A; Cdk1; Cdc25B/Cdc25C phosphatases	Human prostate cancer, LoVo cells, human colon cancer cells, human gastric cancer MGC803, MDA-MB231 human breast cancer cells	[47,58,60,62,67,68]
	Silibinin	**Intrinsic cell death pathway**	Bcl-2, PI3K pathway	Human breast cancer call (MCF-7, T47D), glioma cells, ovarian cancer cells, in melanoma cells, in pharynx squamous cell carcinoma, in colon cancer cells and in cervical cancer cells.	[57,58,84,85,94,95,96]
**Activation**	Silibinin	**G1 phase**	Cdk inhibitors: Kip1/p27, Cip1/p21 and p18/INK4C	Breast cancer cells MDA-MB 468, human prostate cancer PC3 cells, human hepatocellular carcinoma, human colon carcinoma HT-29 cells, human non-small cell lung cancer cell, human prostate carcinoma DU145 cells, ovarian cancer cells, mouse prostate model, HepG2 cells	[47,48,49,51,52,56,57,64,65,69,70,91,97]
			Rb	Human non-small cell lung cancer cell, Apc (−/+) mice, human prostate carcinoma DU145 cells, HepG2 cells	[52,59,65,70,85]
		**Intrinsic cell death pathway**	Bax protein, capsase-3	Glioma cells	[85]
		**Extrinsic cell death pathway**	TRAIL/TRAIL Death receptor 5 (DR5), DR4, caspase-3, -8, -10	Hepatocarcinoma cells, colon cancer SW480 cells	[98,99]

**Table 3:** Clinical studies of Silymarin and derivatives in a cancer context.

**Compounds**	**Silymarin ± FOLFIRI**	**Identifier**	NCT03130634	**Ref**
		**Cancer type**	Metastatic colorectal cancer and received chemotherapy with FOLFIRI regimen	N/A
		**Study design/type**	Interventional	
		**Sample size and phase**	70 patients (between 20 and 80 years old)/Phase 4 study	
		**Dose/administration route**	Experimental arm: during six cycles of FOLFIRI chemotherapy, silymarin (150 mg) three times daily from day 1 to day 7 during one cycle of treatment Control arm: during six cycles of FOLFIRI chemotherapy, patients did not received silymarin during chemotherapy	
		**Outcome measures**	Silymarin to improve the intestinal side effect of the patients undergoing FOLFIRI chemotherapy	
		**Results**	Not yet available	
	**Oral Green Tea Extract and Milk Thistle Extract**	**Identifier**	NCT01239095	N/A
		**Cancer type**	Colorectal cancer patients undergoing resection	
		**Study design/type**	Interventional, single group assignment	
		**Sample size and phase**	30 patients (between 18 and 85 years old) Phase 1 study	
		**Dose/administration route**	Experimental arm: Green tea extract (3200 mg per day) plus Milk thistle extract with phosphatidylcholine (2700 mg per day) For one week prior to surgery and for 30 days after surgery	
		**Outcome measures**	Number of patients with adverse events or complications (time frame 60 days)	
		**Results**	Not yet available	
	**Silybin formulated with phosphatidylcholine (Siliphos; improves its systemic availability compared with silymarin)**	**Identifier**	R621-IEO661/511	[114]
		**Cancer type**	Breast cancer patients with newly diagnosed breast cancer not eligible for neoadjuvant treatment and candidate for surgical lumpectomy or mastectomy	
		**Study design/type**	Pilot presurgical study	
		**Sample size and phase**	12 consecutive patients (women of 18 years old or older), Phase 1study	
		**Dose/administration route**	Silybin formulated in granules to be suspended in drinkable water. Each sachet contained 2.8 g of Siliphos (containing between 29.7 and 36.3% of silybin). A single sachet once daily for 4 weeks until surgery, in an empty stomach (30 min before eating, at least 2 h after the previous meal)	
		**Outcome measures**	Silybin pharmacokinetic profile and pharmacodynamic effects on malignant as well as surrounding normal tissue	
		**Results**	Silybin levels were measured before (SIL) and after (TOT-SIL) enzymatic hydrolysis by HPLC-MS/MS in biologic samples (plasma, urine, breast cancer and surrounding normal tissue). Despite a high between-subject variability, repeated administration of Siliphos achieved levels of TOT-SIL of 31,121 to 7654 ng/mL in the plasma and up to 1375 ng/g in breast cancer tissue. SIL concentrations ranged from 10,861 to 1818 ng/mL in plasma and up to 177 ng/g in breast cancer tissue. Median TOT-SIL concentration was higher in the tumor as compared with the adjacent normal tissue (*P* = 0.018). No significant change in either blood levels of IGF-I and nitric oxide or Ki-67 in tumors was noted.	
	**Silybin formulated with phosphatidylcholine (Siliphos)**	**Identifier**	FDA approval #107662	[115]
		**Cancer type**	Advanced Hepatocellular Carcinoma patients	
		**Study design/type**		
		**Sample size and phase**	30 patients were supposed to be enrolled in the study but only 3 patients could be included (Male aged of 47, 54 and 60 years old)/Phase 1study	
		**Dose/administration route**	Siliphos powder (1:1 ratio of silybin to phosphatidylcholine, which increases drug absorption). All patients orally received 2g of Siliphos per day over 12 weeks	
		**Outcome measures**	Primary endpoint was to determine maximal tolerated dose (MTD) of Siliphos. The secondary endpoints were to (a) mean intrapatient percentage change in AST, ALT and total serum bilirubin levels; (b) quality of life as measured by the FACT (Functional Assessment of Cancer Therapy)–Hepatobiliary questionnaire; (c) plasma concentrations of silibinin and silibinin glucuronide; (d) mean intrapatient percentage change in serum inflammatory biomarkers; and (e) tumor response as measured by RECIST criteria and α-fetoprotein (AFP) concentrations. Exploratory aims were to evaluate *a*) tumor response as measured by RECIST criteria and AFP concentrations and (*b*) survival at 12 months	
		**Results**	Increased plasma concentrations of silybinin and silibinin glucuronide within 1 to 3 weeks were observed. Only one patient out of 3 showed some improvements in liver function abnormalities and inflammatory biomarkers but after 56 days of intervention. All patients died within 23 to 69 days of enrolling into the trial. No MTD could be determined	
	**Silymarin and selenium combination (SM-Se formulation)**	**Identifier**	N/A	[116]
		**Cancer type**	Prostate cancer patients	
		**Study design/type**	6 months randomized controlled double-blind trial	
		**Sample size and phase**	37 patients (men) 2 to 3 months after radical prostatectomy and aged between 51 to 72 years’ old, Phase 1 study	
		**Dose/administration route**	Experimental arm: SM-Se tablet containing 190 mg silymarin of the following composition (%; w/w): taxifolin 4.13, silychristin 17.00, silydianin 7.70, silibinin A 23.66, silibinin B 29.01, isosilibinin A+B 11.38 and undefined components 7.11; 80 μg selenium as selenomethionine Control arm: Placebo tablet contained microcrystalline cellulose (250 mg), isomalt (250 mg) and hydroxypropyl cellulose (10 mg). Patients received either SM-Se or placebo tablets for 6 months (3 tablets/day)	
		**Outcome measures**	Evaluation of the safety and tolerability of a 6 months’ daily consumption of 570 mg silymarin and 240 µg selenium and evaluation of the efficacy to reduce prostate cancer progression markers	
		**Results**	Physical examination, quality of life score (QoL), hematology, basic clinical chemistry and oxidative stress markers, selenium and testosterone levels, antioxidant status were evaluated at baseline, at 3 and 6 months. Data showed that the combination of silymarin and selenium improved the quality of life (QoL) score, decreased the low-density lipoproteins (LDL) and total cholesterol (markers of prostate cancer progression) and, increased serum selenium levels. The formulation did not show any adverse effects in patients. No improvements were observed in the placebo group.	
	**Silibinin**	**Identifier**	N/A	[117]
		**Cancer type**	Colorectal cancer patients	
		**Study design/type**	Interventional, single group assignment	
		**Sample size and phase**	12 patients (1 female and 11 male) aged between 55 and 78 years’ old with confirmed colorectal carcinoma of stages Dukes A (2 patients), B (5) or C (5), who were to undergo colorectal resection and 12 patients (7 females and 5 males, aged between 49 and 78 years’ old, all Dukes D with hepatic metastatic disease originating from primary colorectal carcinoma, who were to undergo hepatic surgery. One patient who underwent colectomy had preoperative radiotherapy and none preoperative chemotherapy. All, except two patients who underwent hepatic surgery, had received 5-fluorouracil with folinic acid, oxaliplatin and/or irinotecan before recruitment/Phase 1 study	
		**Dose/administration route**	Silibinin was formulated in capsules as silipide (IdB 1016), a phytosome product marketed by Indena SpA. The capsules contained 120 mg of silibinin and soy phosphatidylcholine at a molar ratio of 1:1, constituting in terms of percentage weight ∼40% silibinin and 60% phosphatidylcholine. Patients received silipide at dosages of either 360, 720 or 1440 mg silibinin daily for 7 days before surgery; each daily dose was divided in three equal portions taken in the morning, at noon and in the evening. There were eight individuals per dose level (four patients who underwent colectomy and four who had liver resection). The first and second portions of the first dose were taken at noon and in the evening, respectively, of day 1; the last dose portion was ingested in the morning of day 8 before surgery so that, in total, the seven daily doses were distributed >8 days.	
		**Outcome measures**	Evaluation of silibinin pharmacokinetics and pharmacodynamic parameters. Blood and biopsy samples of normal and malignant colorectum or liver were obtained before dosing, and blood and colorectal or hepatic tissues were collected at resection surgery after the final silipide dose. Levels of silibinin were quantified by high-pressure liquid chromatography-UV, and plasma metabolites were identified by LC-MS. Blood levels of IGFBP-3, IGF-I and the oxidative DNA damage pyrimidopurinone adduct of deoxyguanosine (M1dG) were determined.	
		**Results**	Patients silipide supplementation for 7 days, was safe. Plasma levels of silibinin reached 0.3 to 4 μmol/L, with silibinin monoglucuronide, silibinin diglucuronide, silibinin monosulfate and silibinin glucuronide as major metabolites. Silibin levels in liver and colorectal tissues reached 0.3 to 2.5 nmol/g and 20 to 141 nmol/g, respectively. No significant modifications in plasma levels of IGFBP-3, IGF-1 and M1dG were observed at the end of the intervention.	
	**Silybin-phytosome formulation**	**Identifier**	N/A	[118]
		**Cancer type**	Prostate cancer patients	
		**Study design/type**	Interventional, single group assignment	
		**Sample size and phase**	13 patients (18 years old or older), with histologically confirmed prostate cancer, with progressive disease defined by a rising Prostate-Specific Antigen (PSA) or measurable disease by radiological assessment/Phase 1 study	
		**Dose/administration route**	Silybin-phytosome (Siliphos^®^) formulation obtained from Indena Corporation (Seattle, WA). It is a silibinin and phosphatidylcholine powder containing approximately 30% silibinin by weight, which is mixed with applesauce at the ratio of 1/4 teaspoon of silybin-phytosome to 1 Tablespoon of applesauce. Patients received 3 times a day for 4 weeks the silybin-phytosome formulation. The first daily dose-level was 2.5 g, then 5 g and then increased by increments of 5 g (i.e., 10, 15, 20 g daily); due to the toxicity observed with chronic administration of 15 and 20 g daily, the dose level was reduced to 13 g daily.	
		**Outcome measures**	Evaluation of a high-dose Silybin-phytosome pharmacokinetics in blood and urine samples. Evaluation of the safety and tolerability of the formulation	
		**Results**	For a high dose of the formulation (13 g/day in 3-divided doses), the most notable toxicity observed was gastrointestinal, with grade 1 or 2 unconjugated hyperbilirubinemia observed commonly. The only grade 3 or 4 toxicity noted was one patient with transient grade 3 elevation of Alanine-transaminase (ALT). Silibinin plasma half-life was ranging from 1.79–4.99 h. Interpatient great variability was found notably in urine samples. Silibinin level in urine ranged from undetectable to 28.2 µM. Its mean urine level was found to be 6.4 µM. The mean silibinin-glucuronide level was 253.4 (range of 1.5–982 μM). Maximum tolerated dose (MTD) could not be accurately defined. Finally, no objective PSA responses were found with the formulation.	
	**Silibinin-phytosome formulation (Siliphos) +/- Erlotinib (Tarceva)**	**Identifier**	NCT0214611	N/A
		**Cancer type**	EGFR mutant lung adenocarcinoma patients	
		**Study design/type**	-	
		**Sample size and phase**	42 patients with stage IV lung adenocarcinoma and confirmed EGFR (Epidermal Growth Factor Receptor) mutation, aged between 30 and 80 years’ old, who have not received chemotherapy before or who have received postoperative adjuvant chemotherapy more than 6 months before enrollment/Phase 2 study	
		**Dose/administration route**	Patients group receiving 150 mg/day for 4 weeks of Erlotinib (Tarceva) patients group receiving for 4 weeks 1g/day of Silybin-phytosome. No additional information available	
		**Outcome measures**	The primary endpoint of the study is to evaluate the tumor response rate in patients and secondary endpoint is to evaluate progression-free survival, overall survival and safety of Siliphos	
		**Results**	Not yet available	
